# MYC及BCL-2蛋白双表达对弥漫大B细胞淋巴瘤患者预后影响：倾向性评分匹配分析

**DOI:** 10.3760/cma.j.issn.0253-2727.2022.01.009

**Published:** 2022-01

**Authors:** 景 詹, 诗婕 杨, 薇 张, 道斌 周, 炎 张, 为 王, 冲 魏

**Affiliations:** 中国医学科学院、北京协和医学院北京协和医院血液内科 100730 Department of Hematology, Chinese Academy of Medical Science & Peking Union Medical College, Peking Union Medical College Hospital, Beijing 100730, China

**Keywords:** 淋巴瘤，大B细胞，弥漫性, MYC, BCL-2, 预后, 倾向性评分, Lymphoma, large B cell, diffuse, MYC, BCL-2, Prognosis, Propensity score matching

## Abstract

**目的:**

探究MYC/BCL-2蛋白双表达对弥漫大B细胞淋巴瘤（DLBCL）患者预后的影响，观察纳入DA-EPOCH-R（利妥昔单抗+依托泊苷+泼尼松+长春新碱+环磷酰胺+表阿霉素）、中枢神经系统预防治疗、移植等治疗因素后，双表达是否仍是DLBCL的独立预后不良因素。

**方法:**

回顾性收集2015−2018年在北京协和医院血液科治疗且具有可用病理结果的223例初治DLBCL患者，75例MYC/BCL-2高表达的患者归为双表达组，从148例非双表达的患者中，应用倾向性评分（PSM），根据年龄、国际预后指数（IPI）评分、治疗选择等因素进行1∶1匹配，筛选出75例作为对照组，比较两组患者总生存（OS）及无进展生存（PFS）等方面的差异。

**结果:**

PSM后双表达组和非双表达组的3年OS率分别为（69.8±5.5）％及（77.0±4.9）％（*P*＝0.225），3年PFS率分别为（60.7±5.8）％及（65.3±5.5）％（*P*＝0.390），差异均无统计学意义。R-CHOP方案（利妥昔单抗+环磷酰胺+阿霉素+长春新碱+泼尼松）治疗的亚组分析显示，双表达和非双表达患者的3年OS率分别为（61.3±7.5）％及（77.2±5.6）％（*P*＝0.027），3年PFS率分别为（52.1±7.5）％及（70.6 ± 6.0）％（*P*＝0.040），差异均具有统计学意义。多因素Cox回归分析显示年龄、Ann Arbor分期、细胞起源（COO）分型、是否进行中枢神经系统预防、是否进行移植是DLBCL患者预后的独立影响因素（*P*值均<0.05），而MYC/BCL-2蛋白双表达不是预后的影响因素。

**结论:**

MYC/BCL-2蛋白双表达在R-CHOP方案治疗下与不良预后显著相关，但在DA-EPOCH-R、移植等治疗方案下，双表达对DLBCL的不良预后影响在一定程度上得到消除。

## Abstract (en)

**Objective:**

We investigated the impact of MYC/BCL-2 protein co-expression on the prognosis of diffuse large B-cell lymphoma（DLBCL）patients and observed whether double expression（DE）remains an independent poor prognostic factor in DLBCL after the addition of therapeutic factors such as DA-EPOCH-R, central prophylaxis, and transplantation.

**Methods:**

Available pathological findings were retrospectively collected from 223 DLBCL patients at the Peking Union Medical College Hospital from 2015 to 2018. Seventy-five patients with high MYC/BCL-2 expression were categorized as the DE group. From the 148 non-DE patients, 75 DLBCL patients were selected as the control group, using a 1∶1 matching on propensity scores for age, international prognostic index score, treatment choice, and etc. The differences in overall survival（OS）and progression-free survival（PFS）between the two groups were compared.

**Results:**

The 3-year OS was（69.8±5.5）％ for the DE group and（77.0±4.9）％ for the non-DE group（*P*＝0.225）, while the 3-year PFS was（60.7±5.8）％ and（65.3±5.5）％, respectively（*P*＝0.390）. Subgroup analysis in patients treated with the R-CHOP regimen revealed that for the DE and non-DE patients, the 3-year OS was（61.3±7.5）％ and（77.2±5.6）％（*P*＝0.027）, and the 3-year PFS was（52.1±7.5）％ and（70.6±6.0）％（*P*＝0.040）, respectively. Multivariate analysis showed that age, stage of Ann Arbor, COO staging, whether central prophylaxis was performed, and whether transplantation was performed were significant independent risk factors of the prognosis of DLBCL patients（*P*<0.05）. On the other hand, MYC/BCL-2 protein double expression was not significantly associated with prognostic outcomes.

**Conclusion:**

MYC/BCL-2 protein double expression was significantly associated with poor prognosis under R-CHOP regimen treatment, but the poor prognostic impact of DE on DLBCL was eliminated under intensive regimens such as DA-EPOCH-R and transplantation.

弥漫大B细胞淋巴瘤（DLBCL）是非霍奇金淋巴瘤中最常见的亚型，占所有病例的30％以上[1]。由于DLBCL在病理亚型、基因表达谱等方面具有极高的异质性，不同亚型预后差异明显[2]。其中，同时具有MYC和BCL-2蛋白过表达的DLBCL被称为双表达淋巴瘤，占所有DLBCL患者的18％～34％[3]–[4]。MYC蛋白和BCL-2蛋白与淋巴瘤细胞增殖、抑制凋亡、增加基因组不稳定性等生物学行为相关[5]。多项回顾性研究表明，R-CHOP方案治疗双表达淋巴瘤患者预后极差，5年无进展生存（PFS）和总生存（OS）率均低于40％[3]–[4],[6]。关于双表达淋巴瘤的预后研究大多未纳入治疗相关因素，且回顾性分析中的混杂因素所致偏倚较高。故本回顾性研究中我们通过倾向性评分（PSM）校正双表达和非双表达DLBCL患者的临床特点与治疗选择，探索双表达对预后的影响，现报道如下。

## 病例与方法

1. 病例：回顾性收集2015年1月至2018年12月于北京协和医院初治的286例DLBCL患者临床资料，排除无可用病理结果的52例患者及未在本院接受过诱导治疗的11例患者，最终纳入223例DLBCL患者。所有患者均根据临床症状和组织活检结果，按照WHO 2008年淋巴组织肿瘤分类标准[2]进行诊断。根据免疫组化结果，设定MYC、BCL-2蛋白的阳性截断值分别为40％、50％[5]，MYC与BCL-2蛋白均高表达的75例患者为双表达组，其余148例为非双表达组。临床资料包括性别、年龄、基础疾病史、Ann Arbor分期、国际预后指数（IPI）评分、化疗前外周血LDH水平、细胞起源（COO）分型、治疗选择等。其中基础疾病包括高血压、糖尿病、冠心病等慢性全身性疾病或乙型肝炎、EB病毒（EBV）等感染性疾病。

2. 治疗方案：初始诱导治疗：①R-CHOP方案（21 d）：利妥昔单抗（375 mg/m^2^，第1天）、环磷酰胺（750 mg/m^2^，第1天）、阿霉素（50 mg/m^2^，第1天）、长春新碱（1.4 mg/m^2^，第1天）和泼尼松（100 mg/d^1^，第1～5天）；②DA-EPOCH-R方案（21 d）：利妥昔单抗（375 mg/m^2^，第1天）、依托泊苷（50 mg·m^−2^·d^−1^，第1～4天）、泼尼松（60 mg/m^2^每日2次，第1～5天）、长春新碱（0.8 mg·m^−2^·d^−1^，第1～4天）、环磷酰胺（750 mg/m^2^，第5天）和表阿霉素（15 mg·m^−2^·d^−1^，第1～4天）（剂量调整参照既往研究[7]）。中枢神经系统（CNS）预防治疗指1次以上阿糖胞苷和地塞米松鞘内注射或甲氨蝶呤联合诱导治疗，适用于肿瘤在特定部位的患者，包括副鼻窦、硬膜外组织、骨髓、睾丸、乳腺、肾上腺、女性生殖器官、皮肤以及有2个以上的体外病灶。

3. 随访：通过查阅患者门急诊及住院病历、电话回访的方式进行随访。随访截止日期为2021年4月10日，中位随访47.1个月。OS期定义为诊断日期至死亡或末次随访时间。PFS期定义为治疗开始日期至第1次复发/疾病进展日期、因淋巴瘤导致死亡日期或末次随访日期（无复发患者）。

4. 免疫组化：由两位经验丰富的形态学和病理学专家对所有石蜡样本重新染色，并独立进行重新分析，以获得准确的免疫组化结果。此外，利用Hans算法分析CD10、BCL-6和MUM-1蛋白的免疫组化结果[8]，COO分型确定生发中心B细胞样（GCB）和非GCB亚型。

5. 疗效及不良反应评估：按照2007年国际工作组标准[9]，通过腰椎穿刺、PET-CT、增强CT评估治疗效果和疾病进展情况。在初始治疗第3～4个疗程后及治疗结束后分别进行影像学评价。治疗过程中的不良反应根据《不良事件通用术语标准》（CTCAE）v5.0进行评价。治疗相关死亡事件定义为因感染或大量出血而非疾病进展而死亡。

6. 统计学处理：使用SPSS 25.0统计学软件进行分析。根据性别、年龄、基础疾病史、Ann Arbor分期、IPI评分、LDH水平、COO分型、治疗方案、CNS预防、放疗、移植等指标，对75例MYC/BCL-2双表达患者及148例非双表达患者按1∶1进行PSM匹配，卡钳值设为0.1；分类资料组间比较采用卡方检验或Fisher精确概率法，生存分析采用Kaplan-Meier 法，组间比较采用Log-rank检验，风险比（*HR*）通过Cox比例风险模型计算。除IPI外，单因素分析中*P*<0.15的因素纳入多因素分析，多因素Cox分析采用条件后退法，*P*<0.05为差异有统计学意义。

## 结果

1. PSM结果：75例双表达组患者及148例非双表达组的临床特征及治疗选择资料见表1。其中两组仅在治疗方案方面的差异有统计学意义（*P*＝0.048），而性别、年龄、有无基础疾病史、Ann Arbor分期、IPI评分、LDH水平等方面的差异无统计学意义。应用PSM根据性别、年龄、有无基础疾病史、Ann Arbor分期、IPI评分、LDH水平、COO分型、诱导化疗方案、CNS预防应用情况、移植、放疗等临床因素，按1∶1进行匹配，选择出75例非双表达的患者作为对照组，匹配后两组患者临床基线特征与治疗选择方面的差异均无统计学意义，具有可比性（表1）。

**表1:** 倾向性评分（PSM）前后双表达组和非双表达组DLBCL患者临床基线特征与治疗选择比较［例数（％）］

临床特征及治疗选择	PSM前			PSM后		
	双表达组（75例）	非双表达组（148例）	*P*值	双表达组（75例）	非双表达组（75例）	*P*值
性别			0.855			0.414
男	34（45.3）	69（46.6）		34（45.3）	39（52.0）	
女	41（54.7）	79（53.4）		41（54.7）	36（48.0）	
年龄			0.385			0.324
<60岁	39（52.0）	86（58.1）		39（52.0）	45（60.0）	
≥60岁	36（48.0）	62（41.9）		36（48.0）	30（40.0）	
基础疾病			0.697			0.410
无	40（53.3）	83（56.1）		40（53.3）	45（60.0）	
有	35（46.7）	65（43.9）		35（46.7）	30（40.0）	
Ann Arbor分期			0.870			0.570
Ⅰ~Ⅱ期	17（22.7）	35（23.6）		17（22.7）	20（26.7）	
Ⅲ~Ⅳ期	58（77.3）	113（76.4）		58（77.3）	55（73.3）	
IPI评分			0.572			0.744
0～2分	35（46.7）	75（50.7）		35（46.7）	37（49.3）	
3～5分	40（53.3）	73（49.3）		40（53.3）	38（50.7）	
LDH水平			0.347			0.325
≤250 U/L	31（41.3）	71（48.0）		31（41.3）	37（49.3）	
>250 U/L	44（58.7）	77（52.0）		44（58.7）	38（50.7）	
COO分型			0.155			0.734
GCB型	26（34.7）	66（44.6）		26（34.7）	28（37.3）	
非GCB型	49（65.3）	82（55.4）		49（65.3）	47（62.7）	
MYC表达			<0.001			<0.001
<40％	0（0.0）	120（81.1）		0（0.0）	59（78.7）	
≥40％	75（100.0）	28（18.9）		75（100.0）	16（21.3）	
DHL			0.706			0.344
无	45（93.7）	100（95.2）		45（93.7）	52（98.1）	
有	3（6.3）	5（4.8）		3（6.3）	1（1.9）	
不详	27	43		27	22	
治疗方案			0.048			0.104
R-CHOP	49（60.0）	115（77.3）		49（65.3）	58（77.3）	
DA-EPOCH-R	26（34.7）	33（22.3）		26（34.7）	17（22.7）	
CNS预防			0.481			0.702
无	17（22.7）	40（27.0）		17（22.7）	19（25.3）	
有	58（77.3）	108（73.0）		58（77.3）	56（74.7）	
造血干细胞放疗			0.702			0.719
无	70（93.3）	136（91.9）		70（93.3）	72（96.0）	
有	5（6.7）	12（8.1）		5（6.7）	3（4.0）	
有无移植			0.128			0.220
无	68（90.7）	123（83.1）		68（90.7）	63（84.0）	
有	7（9.3）	25（16.9）		7（9.3）	12（16.0）	

注：DLBCL：弥漫大B细胞淋巴瘤；IPI评分：淋巴瘤国际预后指数评分；LDH：乳酸脱氢酶；GCB：生发中心来源；DHL：双打击淋巴瘤；R-CHOP：利妥昔单抗+环磷酰胺+阿霉素+长春新碱+泼尼松；DA-EPOCH-R：利妥昔单抗+依托泊苷+泼尼松+长春新碱+环磷酰胺+表阿霉素；CNS：中枢神经系统

2. PSM后两组近期疗效及生存情况比较：初始治疗结束后进行疗效评价，75例双表达患者完全缓解（CR）42例（56.0％），75例非双表达患者CR 43例（57.3％），差异无统计学意义（*P*＝0.869）。两组3年OS率分别为（69.8 ± 5.5）％、（77.0 ± 4.9）％（*χ*^2^＝1.472，*P*＝0.225），差异无统计学意义（图1A）；3年PFS率分别为（60.7 ± 5.8）％、（65.3 ± 5.5）％（*χ*^2^＝0.739，*P*＝0.390），差异也无统计学意义（图1B）。

**图1:**
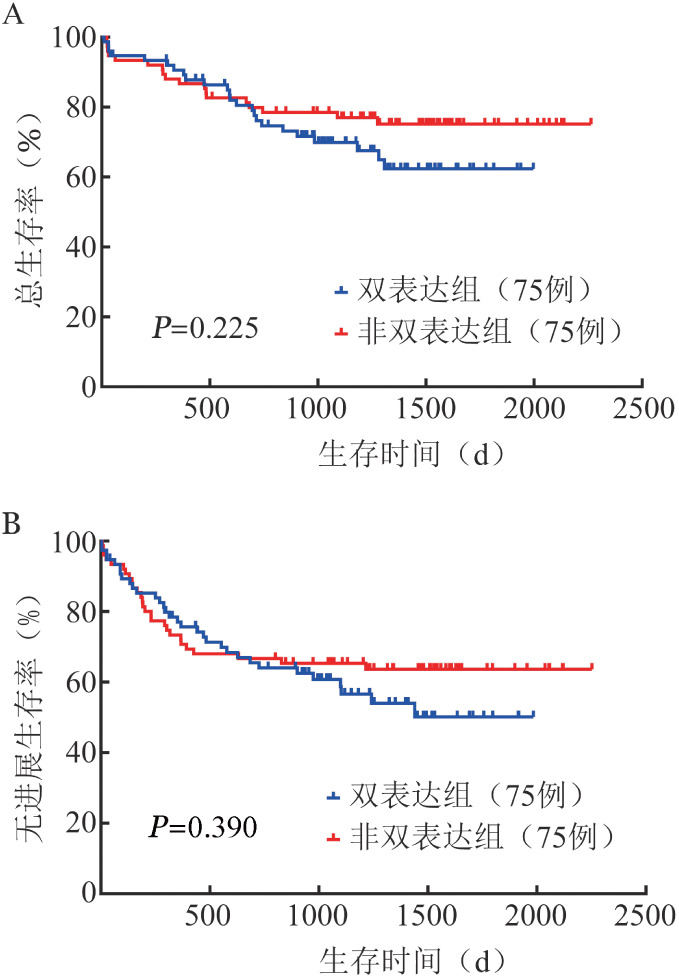
MYC/BCL-2双表达与非双表达弥漫大B细胞淋巴瘤（DLBCL）患者总生存（A）及无进展生存（B）曲线

3. PSM后R-CHOP治疗亚组中双表达与非双表达生存对比：进一步对两组中接受R-CHOP方案治疗的患者进行亚组分析，分别有49例双表达患者与58例非双表达患者接受R-CHOP方案治疗，其中25例（51.0％）双表达患者和35例（60.3％）非双表达患者达到CR，差异无统计学意义（*χ*^2^＝0.938，*P*＝0.333）。两组3年OS率分别为（61.3 ± 7.5）％、（77.2 ± 5.6）％（*χ*^2^＝4.892，*P*＝0.027）（图2A）；3年PFS率分别为（52.1 ± 7.5）％、（70.6 ± 6.0）％（*χ*^2^＝4.201，*P*＝0.040）（图2B）。R-CHOP方案治疗下双表达与非双表达两组的3年OS率、PFS率的差异均有统计学意义。

**图2:**
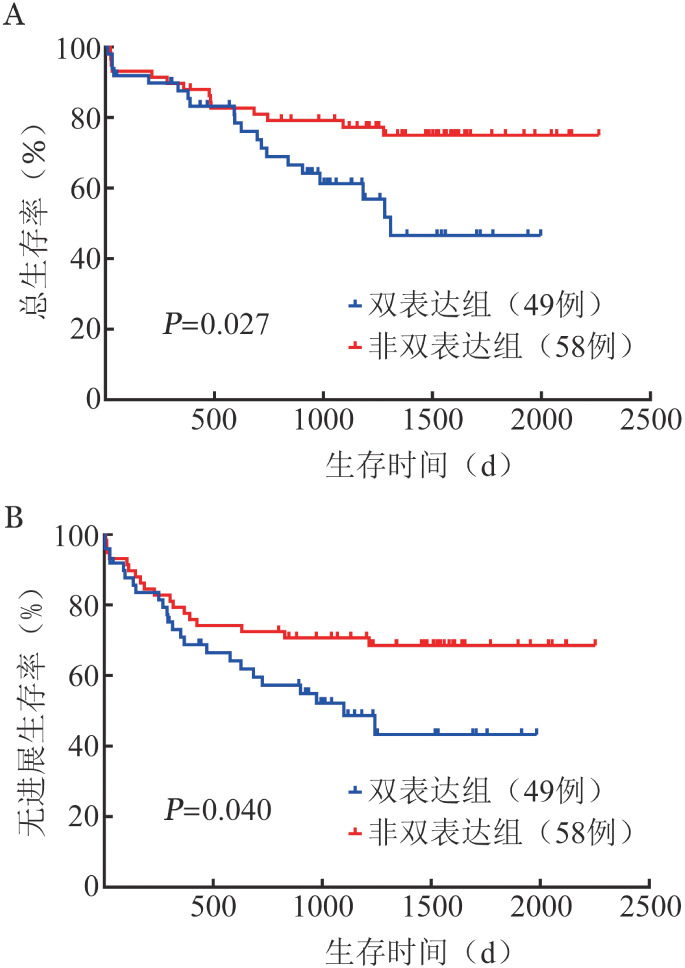
R-CHOP治疗方案下MYC/BCL-2双表达与非双表达DLBCL患者总生存（A）及无进展生存（B）曲线 R-CHOP：利妥昔单抗+环磷酰胺+阿霉素+长春新碱+泼尼松；DLBCL：弥漫大B细胞淋巴瘤

4. PSM后DLBCL患者预后影响因素分析：结果如表2所示，单因素Cox分析显示，与预后相关的潜在因素是年龄、疾病分期、IPI指数、LDH水平、COO分型、MYC蛋白表达、是否进行CNS预防、是否进行移植。将单因素分析中*P*<0.15的因素纳入多因素Cox回归分析，结果显示年龄、Ann Arbor分期、COO分型、是否进行CNS预防、是否进行移植是影响DLBCL患者预后的独立影响因素（*P*<0.05）。而MYC和BCL-2蛋白双表达无论在单因素还是多因素Cox回归分析中均不是预后的影响因素。

**表2:** 倾向性评分（PSM）后弥漫大B细胞淋巴瘤患者预后的单因素及多因素Cox回归分析

因素	无进展生存						总生存					
	单因素分析			多因素分析			单因素分析			多因素分析		
	*HR*	95％ *CI*	*P*值	*HR*	95％ *CI*	*P*值	*HR*	95％ *CI*	*P*值	*HR*	95％ *CI*	*P*值
女性	1.172	0.702～1.957	0.543				0.952	0.520～1.743	0.873			
≥60岁	1.863	1.114～3.116	0.018	1.834	1.092～3.080	0.022	2.179	1.176～4.037	0.013	2.078	1.109～3.893	0.022
有基础疾病	1.323	0.794～2.206	0.282				1.055	0.574～1.937	0.863			
Ann ArborⅢ～Ⅳ期	2.547	1.208～5.369	0.014	2.999	1.418～6.342	0.004	5.467	1.688～17.707	0.005	7.622	2.322～25.017	0.001
IPI 3～5分	1.963	1.157～3.329	0.012				2.372	1.247～4.510	0.008			
LDH>250 U/L	1.802	1.057～3.073	0.030				1.745	0.927～3.282	0.084			
非GCB型	2.019	1.123～3.631	0.019	2.235	1.237～4.037	0.008	1.770	0.890～3.523	0.104	1.841	0.919～3.692	0.085
非双表达	1.252	0.749～2.093	0.391				1.458	0.790～2.691	0.228			
MYC≥40％	1.055	0.625～1.782	0.840				1.250	0.664～2.352	0.489			
DA-EPOCH-R方案^a^	1.002	0.570～1.760	0.995				0.530	0.245～1.144	0.106			
有CNS预防	0.694	0.391～1.233	0.213				0.591	0.307～1.137	0.115	0.439	0.225～0.857	0.016
放疗	0.899	0.281～2.872	0.857				0.408	0.056～2.966	0.376			
移植	0.184	0.045～0.753	0.019	0.137	0.033～0.564	0.006	0.038	0.001～1.503	0.082			

注：IPI：国际预后指数；GCB：生发中心来源；DA-EPOCH-R：利妥昔单抗+依托泊苷+泼尼松+长春新碱+环磷酰胺+表阿霉素；CNS：中枢神经系统。a：与R-CHOP方案（利妥昔单抗+环磷酰胺+阿霉素+长春新碱+泼尼松）比较

5. PSM后非双表达组内MYC高表达与低/表达生存对比：进一步对非双表达组中16例MYC高表达和59例MYC低/无表达的患者进行亚组分析，其中10例（62.5％）MYC高表达患者和33例（55.9％）低/无表达患者达CR，差异无统计学意义（*χ*^2^＝0.222，*P*＝0.638）。两组3年OS率分别为（81.3±9.8）％、（75.7±5.7）％（*χ*^2^＝0.217，*P*＝0.641），差异无统计学意义；3年PFS率分别为（75.0±10.8）％、（62.7±6.3）％（*χ*^2^＝0.618，*P*＝0.432），差异也无统计学意义。

6. PSM后DA-EPOCH-R方案与R-CHOP方案不良反应比较：共对107例接受R-CHOP治疗的患者和43例接受DA-EPOCH-R治疗的患者进行不良反应评估。与DA-EPOCH-R治疗的患者相比，R-CHOP治疗的患者3/4级中性粒细胞减少（37.4％对65.1％，*P*＝0.001）、贫血（11.2％对20.9％，*P*＝0.121）和血小板减少（14.0％对30.2％，*P*＝0.021）发生率更低。然而，R-CHOP和DA-EPOCH-R方案中出现粒细胞缺乏伴发热事件的发生率相近（20.6％对27.9％，*P*＝0.331）。R-CHOP和DA-EPOCH-R方案组分别有3例（2.8％）和2例（4.7％）患者发生治疗相关死亡事件（*P*＝0.625）。

## 讨论

近来随着对DLBCL分子异质性认识的不断加深，MYC和BCL-2蛋白在DLBCL中发挥的作用也逐渐明确。MYC原癌基因位于染色体8q24上，其编码的转录因子在能量代谢、蛋白质合成、细胞分化等方面具有重要作用。MYC蛋白异常表达可由染色体易位、MYC基因扩增、基因内突变和拷贝数改变等多种机制引起，其中最常见的遗传事件为MYC与免疫球蛋白基因（IG）位点的转位突变[10]–[11]。淋巴瘤细胞中MYC蛋白表达增高将导致基因组不稳定、基因扩增和细胞增殖[12]。BCL-2基因也是一种位于染色体18q21的原癌基因，其编码的BCL-2蛋白具有抑制细胞凋亡、维持细胞增殖的功能。尤其在同时存在MYC蛋白高表达的情况下，BCL-2蛋白可发挥促进淋巴瘤进展，造成耐药[13]–[14]。BCL-2蛋白增加则通常与核因子κB信号通路的激活有关[15]。

基于MYC蛋白与BCL-2蛋白在DLBCL发生发展中的重要作用，R-CHOP方案治疗下MYC与BCL-2蛋白高表达的预后不良问题已得到广泛重视。如Green等[6]在193例DLBCL患者的回顾性研究中发现双表达患者更易出现体力活动状态差、疾病晚期、Ki-67增殖指数高、IPI评分中/高危、更多结外病灶受累等情况。另外也有多项研究报道DLBCL中MYC/BCL-2共表达与侵袭性临床过程相关，在ABC亚型中更常见，并导致R-CHOP治疗下DLBCL患者总体预后较差[4]–[5]。但目前绝大多数MYC/BCL-2双表达淋巴瘤的预后研究均为回顾性研究，混杂因素所致偏倚较高，且并未纳入治疗相关因素进行分析，因此DA-EPOCH-R的强化疗方案、CNS预防治疗、放疗、化疗对于双表达淋巴瘤患者预后的影响仍未完全明确。对于使用DA-EPOCH-R、进行移植治疗的部分DLBCL患者，双表达是否仍是此类患者的预后不良因素也尚未查阅到相关报道。

由于回顾性分析并未采用前瞻性随机对照研究的随机分组方法，无法基于大数定理，消除研究组间的数据偏差及混杂变量的影响，因此回顾性研究极易出现系统性偏差。而PSM作为一种可用于利用非实验数据进行干预效应分析的统计学方法，可在一定程度上消除组别之间的干扰因素。本研究中我们回顾性纳入2015年1月至2018年12月于北京协和医院初治的DLBCL患者共223例进行分析。其中，75例（33.6％）为双表达组，与既往研究相符（18％～34％）[3]–[4]。随后对两组患者的临床基线特征与治疗选择比较，发现两组间在初始治疗方案选择方面的差异有统计学意义（*P*＝0.048），于是利用PSM进行1∶1匹配，消除了部分双表达组、非双表达组患者之间的选择性偏倚，使其资料更具可比性，提高了分析结果的可信度。

本研究结果显示，在仅使用R-CHOP治疗的患者中，MYC/BCL-2蛋白高表达组与非高表达组患者的OS和PFS率差异有统计学意义，与既往国内外研究结果[4]–[6]相符。但在纳入DA-EPOCH-R治疗、移植、CNS预防等临床治疗因素之后，MYC/BCL-2蛋白双表达与DLBCL患者3年的OS和PFS率差异无统计学意义，不再是DLBCL的独立预后因素。提示在DA-EPOCH-R、移植等治疗方案下，双表达对DLBCL的不良预后影响可能在一定程度上得到消除。

DA-EPOCH-R方案已在双表达淋巴瘤（具有MYC基因扩增或易位，同时伴BCL-2基因扩增或易位和/或BCL6基因扩增或易位的DLBCL）、复发或难治性DLBCL以及某些与高侵袭性、高增殖相关的DLBCL亚组患者中表现出了良好的效果[16]–[17]。但在双表达淋巴瘤患者中DA-EPOCH-R方案的治疗效果存在一些争议性的发现。2017年刘薇等[18]证实DA-EPOCH-R方案在MYC/BCL-2蛋白双表达淋巴瘤患者的一线治疗中具有良好的疗效。2019年Dodero等[19]对114例患者的随访研究中发现，DA-EPOCH-R治疗方案可显著提升65岁以下双表达淋巴瘤患者的PFS和OS率，但65岁以上患者无明显获益。同年Zhang等[20]在另一项纳入了189例DLBCL患者的回顾性研究中得出，DA-EPOCH-R方案不改善双表达淋巴瘤患者的预后，仅可使60岁以下、GCB亚型、高危IPI的部分患者获益，并且双表达仍是DLBCL的独立预后不良因素。此外，有其他研究证明自体造血干细胞移植或异基因造血干细胞移植后，相比于无MYC/BCL-2蛋白同时高表达的患者，双表达患者的预后仍显著差于非双表达患者，双表达仍是DLBCL的独立不良预后因素[21]–[22]。由此可见，目前关于MYC/BCL-2蛋白双表达淋巴瘤患者的治疗方案仍然充满争议，需要更多前瞻性研究探索。

此外，DA-EPOCH-R方案的安全性也是临床应用的前提。临床使用过程中必须综合考虑年龄、合并症、对初始化疗的耐受性、ECOG评分、骨髓功能等因素方可进行个体化治疗，尤其是对于基础情况较差的老年DLBCL患者[23]。本研究结果显示，虽然DA-EPOCH-R方案治疗下患者的血液学不良反应很常见，但粒细胞缺乏伴发热事件的发生率和治疗相关死亡率并未较R-CHOP方案显著增加，这可能与临床中接受DA-EPOCH-R方案的患者更积极应用预防性抗生素和G-CSF有关。

总之，本研究结果显示在DA-EPOCH-R、移植、CNS预防等治疗下，MYC/BCL-2蛋白双表达不再是DLBCL的独立危险因素，其不良预后影响在一定程度上得到消除。但本研究也存在一些不足：①本研究为回顾性研究，尽管已使用PSM，相较于前瞻性实验仍不能完全控制偏倚；②病例数较为有限；③中位随访时间未达到5年。因此，本研究结论需要大规模前瞻性研究来进一步验证。同时我们将继续跟踪调查长期生存结果，并在未来的研究中增加样本量进行分析。
